# Application of aptamer functionalized nanomaterials in targeting therapeutics of typical tumors

**DOI:** 10.3389/fbioe.2023.1092901

**Published:** 2023-02-16

**Authors:** Xiujuan Yin, Zhenqiang He, Weiying Ge, Zhenhua Zhao

**Affiliations:** ^1^ Department of Radiology, Shaoxing People’s Hospital, Shaoxing, China; ^2^ Key Laboratory of Functional Molecular Imaging of Tumor and Interventional Diagnosis and Treatment of Shaoxing City, Shaoxing, China; ^3^ Clinical Medical College of Hebei University, Baoding, China; ^4^ Department of Radiology, Hebei University Affiliated Hospital, Baoding, China; ^5^ Medical College of Zhejiang University, Hangzhou, China

**Keywords:** aptamer, nanomaterials, targeting therapeutics, tumours, drug delivery

## Abstract

Cancer is a major cause of human death all over the world. Traditional cancer treatments include surgery, radiotherapy, chemotherapy, immunotherapy, and hormone therapy. Although these conventional treatment methods improve the overall survival rate, there are some problems, such as easy recurrence, poor treatment, and great side effects. Targeted therapy of tumors is a hot research topic at present. Nanomaterials are essential carriers of targeted drug delivery, and nucleic acid aptamers have become one of the most important targets for targeted tumor therapy because of their high stability, high affinity, and high selectivity. At present, aptamer-functionalized nanomaterials (AFNs), which combine the unique selective recognition characteristics of aptamers with the high-loading performance of nanomaterials, have been widely studied in the field of targeted tumor therapy. Based on the reported application of AFNs in the biomedical field, we introduce the characteristics of aptamer and nanomaterials, and the advantages of AFNs first. Then introduce the conventional treatment methods for glioma, oral cancer, lung cancer, breast cancer, liver cancer, colon cancer, pancreatic cancer, ovarian cancer, and prostate cancer, and the application of AFNs in targeted therapy of these tumors. Finally, we discuss the progress and challenges of AFNs in this field.

## Graphical Abstract

**Figure:**
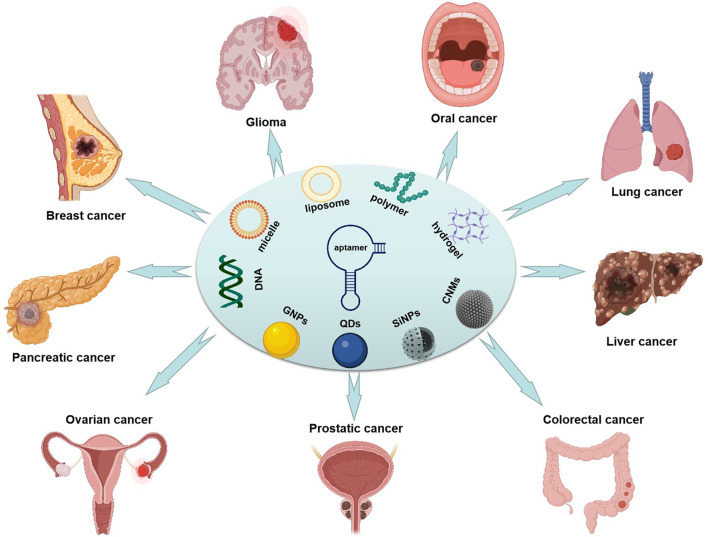
Illustrated the application of aptamer functionalized nano-materials in typical tumors.

## Introduction

Accurate diagnostics and effective therapeutics are the keys to the prognosis of diseases. It is always an important research hotspot to develop drugs targeting tumor cells to realize precise therapeutics. Among many substances, aptamers are considered one of the drugs with potential application value due to their unique properties. Aptamers were first proposed by Gold and Tuerk in 1990. They are short functional single-stranded DNA or RNA molecules, usually consisting of 20–80 nucleotides, which are folded into different secondary and tertiary structures by hydrogen bonding, van der Waals force, or electrostatic interaction (Tuerk and Gold, 1990). Each unique sequence contains random bases (20–50 nt) with two conserved primer binding sites on both sides, which are used for the PCR amplification step. In the process of selection, the library is incubated, and in the process of selection, the library is incubated with the purified target molecule for a specific period of time. Then the unbound sequence is eliminated (segmentation step), and the target binding sequence is separated, which is directly amplified by PCR (DNA SELEX) or reverse transcribed into an amplifiable cDNA, and then transcribed back to RNAby T7 RNA polymerase (RNA SELEX) (Tuerk and Gold, 1990; Urak et al., 2016). PCR products were used for the next screening. After several rounds of screening, the enriched sequence was sequenced, and its binding ability was further evaluated. After SELEX, the aptamer sequence can be modified to improve its binding affinity and selectivity, thus reducing the off-target effect (Yang et al., 2007). Cell-SELEX is an improvement on the traditional SELEX process, which uses whole living cells as target cells instead of isolated target molecules. Specifically, the oligonucleotide library is incubated with target cells, unbound sequences are removed by washing, and bound sequences are collected at the same time. These sequences can bind to specific molecules on the cell surface (positive selection). After incubating with “negative/non-cancerous” cells, a specific aptamer (negative selection) is further selected, and the selected aptamer is used in the next round of classical SELEX selection *in vitro*. The purified protein has obvious advantages in the screening process and can obtain the best enrichment. For example, when the target is “unknown” or the cell type is specific, the cell-SELEX method is preferred. It is worth noting that in some cases, the target protein may be partially hidden and/or inaccessible in the body, so the whole living cell represents a more physiological state. Because most cancer cells express highly specific surface markers for diagnosis and treatment, cell-SELEX technology plays an important role in cancer biology. Due to this method, the development of aptamers for specific cancer cell receptors/proteins is particular, which creates opportunities for targeted and personalized therapy (Shangguan et al., 2006; Guo et al., 2008).

The characteristics of aptamers are that they can bind various targets specifically (Iliuk et al., 2011) and with high affinities, such as inorganic mental ions (Qu et al., 2016), small organic molecules (Pfeiffer and Mayer, 2016), polypeptides (Agyei et al., 2018), protein (Srivastava et al., 2021), intact living cells (Liu et al., 2022a) and even tissues (Pang et al., 2018). The specificity of aptamers is mainly because they can be folded to form a spiral and single-stranded ring structures (Autiero et al., 2018; Afrasiabi et al., 2020), and the high affinity of aptamers is mainly attributed to non-covalent interactions (Van der Waals forces, hydrogen bonds, and stacking) (Padmanabhan et al., 1993; Huang et al., 2003; Long et al., 2008). In addition, aptamers are also called “chemical antibodies” because of their similar specificity and affinity to protein antibodies. Compared with protein antibodies, aptamers have the advantages of small size, synthesis rapidly, flexible structure, easy chemical modification, good tissue permeability, high chemical stability, easy storage and transportation, no toxicity, no immunogenicity, and so on (Zhang L. et al., 2017; Zhou and Rossi, 2017; Nakatsuka et al., 2018; Wu et al., 2018). These characteristics make aptamers widely used in molecular biology, biosensing, and biomedicine, especially in the fields of diagnostics and therapeutics. Compared with antibodies, the plasma half-life of aptamers are shorter, only a few minutes (Griffin et al., 1993; Hicke et al., 2006). The main reason is that aptamers are sensitive to nuclease, easy to be metabolized and degrade, small in size, and easy to be excreted through the kidney (Panigaj et al., 2019). Therefore, the clinical application of aptamers is developing slowly, and the short half-life of plasma is the main problem of the clinical application of aptamers. One of the solution to prolong the plasma half-life of aptamers and slow down renal excretion are to combine aptamers with microspheres (Carrasquillo et al., 2003), nanoparticles (NPs) (Jo and Ban, 2016), lipid nanoparticles (LNPs) (Li et al., 2020), polymers (Pednekar et al., 2012)and other nanomaterials (Eilers et al., 2020). Therefore, the rapid development of materials science provides new opportunities for aptamers to be applied in biomedicine.

With the rapid development of nanotechnology, many nanomaterials with unique physical and chemical properties have been used in biomedical fields (Yang et al., 2015; Beg et al., 2017; Zhao et al., 2017; Dong et al., 2020; Saghatchi et al., 2020). The development of many nanomaterials has accelerated the progress of diagnostics and therapeutics of related diseases, showing a good prospect for clinical applications (Liu et al., 2022b). Nanomaterials possess ideal physical and chemical properties, including physical adsorption, chemical catalysis, large surface area, good biocompatibility, and stability (Pavlov et al., 2004; Lu et al., 2007). However, most nanomaterials have some disadvantages, such as dosage administration and lack of selective targeting ability, and some nanomaterials have toxic side effects on non-target tissues or organs, all of which limit the development of nanomaterials in biological applications. To overcome these defects of nanomaterials and expand their application *in vivo*, people have adopted different methods to modify nanomaterials onto biomolecules to improve their selective targeting ability (Cansiz et al., 2015; Sivaram et al., 2018).

Immobilization of functional aptamer on the carrier of nanomaterials is a powerful way to construct new multifunctional materials with ideal properties. Aptamers are single-stranded nucleic acid sequences, which show selective binding characteristics to low-molecular-weight nanomaterials and can be functionalized with nanomaterials through covalent or non-covalent interactions (Chiang et al., 2008; Guo et al., 2011). However, the intermolecular interaction, site, and mode of action are the key factors in constructing multifunctional nanomaterials. Nan Liu’s team explored the binding characteristics of aptamers and compounds (Zhang et al., 2019), the interaction between carbon nanotubes and aptamers (Liu et al., 2020a), and established a new evaluation method for molecular beacon binding system (Jia et al., 2017), which laid a certain foundation in the research of aptamer-functionalized nanomaterials. The system based on AFNs composites not only has the dual characteristics of aptamer and nanomaterials, but also has new characteristics such as high affinity, strong degradation, and high cell uptake between functionalized nano-materials and targets. AFNs has large specific surface area, easily accessed, conformable flexibility, high affinity and specificity and biocompatibility, which makes it play a key role in biomedical applications. For example, effective self-delivery to cancer cells is the first condition to explore the intracellular environment (Goud et al., 2019; Qing et al., 2020). In this paper, the research progress of AFNs potential in targeted therapy of typical tumors is reviewed, and the progress and challenges of AFNs in this field are discussed.

## Application of aptamer functionalized nanomaterials in therapeutics

Cancer is a major cause of human death all over the world. It is estimated that there are 19.3 million new cases every year. Traditional methods of therapeutics includes surgery, immunotherapy, hormone therapy and comprehensive therapy (radiotherapy and chemotherapy) (Yingchoncharoen et al., 2016). For cancer patients, drug treatment is an indispensable part of prolonged treatment. Common antitumor drugs include cytotoxic drugs, hormonal drugs, biological response regulators, monoclonal antibody drugs, adjuvants and so on (Zhan et al., 2020). These traditional antitumor drugs have improved the overall survival rate. However, there are still a series of problems, such as easy recurrence, poor therapeutic effects and severe side effects. Therefore, developing an improved drug delivery strategy has always been an important research goal. Aptamers functionalized nanomaterials have become a research hotspot in biomedical fields because of their specific targeted delivery capability. We will focus on the latest experimental research and progress of aptamer-nanodelivery systems in several common tumor diseases.

### Glioma

**FIGURE 1:**
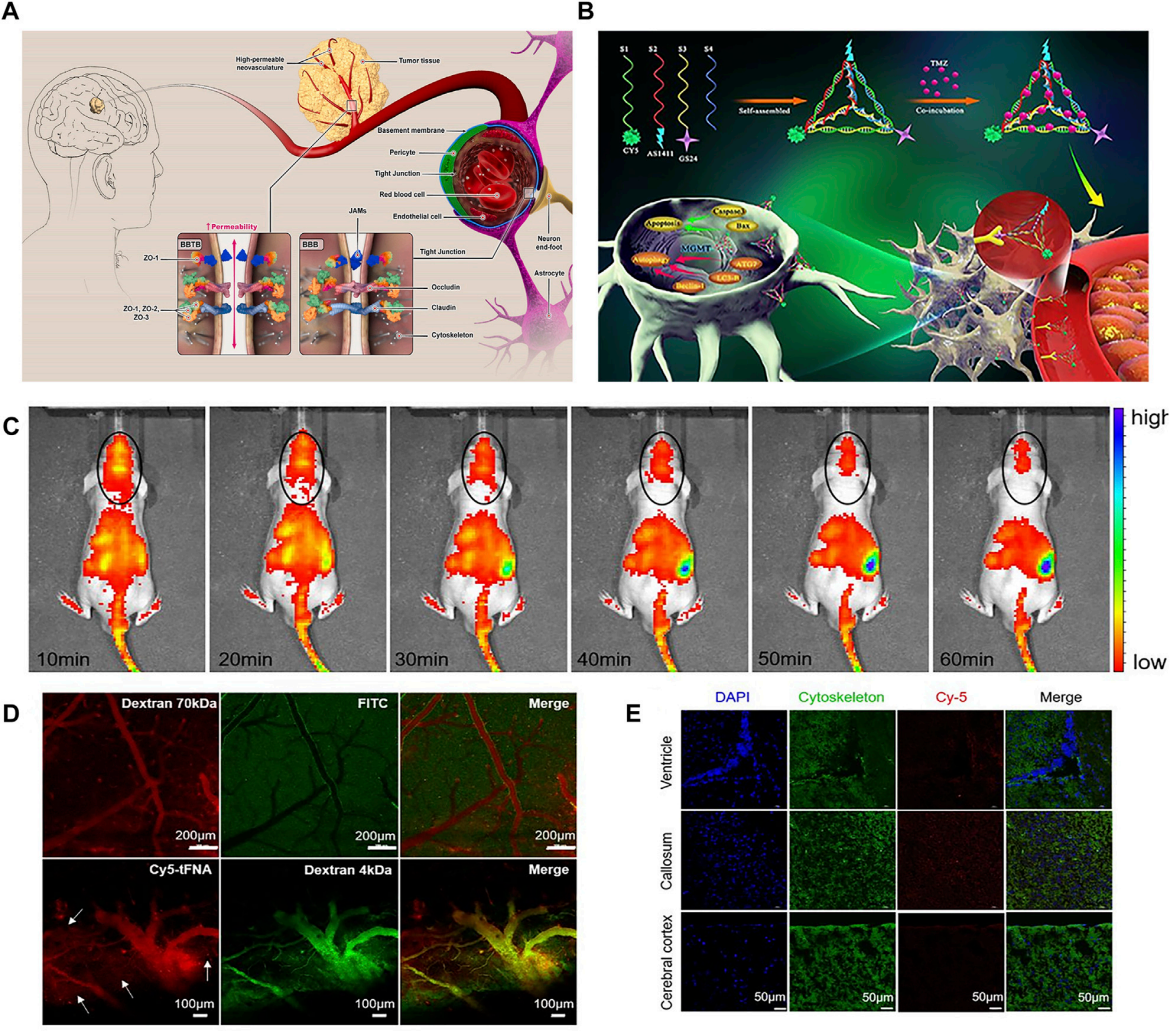
**(A)** schematic concept of structure and function of BBB and BBTB. **(B)** Synthesis of GS24 aptamer tFNA-TMZ nanoparticles and schematic diagram of tFNA-TMZ crossing BBB into GBM cells for treatment. **(C)** *In vivo* fluorescent signal images. **(D)** Biphoton FI imaging showed that Dextran 70 kDa only existed in the vascular lumen. Dextran 4 kDa and tFNA (white arrows) can permeated cerebral vascular wall and infiltrate into the surrounding parenchyma. **(E)** The distribution of tFNA in the different brain area including callosum, ventricle and cerebral cortex. Panel **(A)** was reproduced with permission from Ref. (Mojarad-Jabali et al., 2021), Copyright 2021, Elsevier. Panel **(B–E)** was reproduced with permission from Ref. (Fu et al., 2019), Copyright 2019, American Chemical Society.).

Glioma is a heterogeneous central nervous system (CNS) tumor, originating from glial cells (Lin et al., 2020), accounting for about 30% of all brain tumors and 80% of all malignant primary brain cancers. Moreover, glioblastoma multiforme (GBM) is the most common aggressive glioma. GBM has high proliferation rate, diffuse infiltration, angiogenesis capacity and intra-tumor variability, accounting for 54.7% of all gliomas (Luiz et al., 2021). The global annual incidence rate is estimated to be 3.22 cases per 100,000 people (Chen et al., 2021), and the median survival time is 14 months (Hays et al., 2017). The treatment methods of GBM includes surgical resection, chemotherapy, radiotherapy or comprehensive treatmen (Ozdemir-Kaynak et al., 2018). It is difficult to clearly distinguish normal tissue from tumor tissue by surgical resection, and there is also the risk of brain injury. Due to the existence of blood brain barrier (BBB) and blood brain tumor barrier (BBTB), the permeability of anticancer drugs is poor (Mojarad-Jabali et al., 2021) (Figure 1A), and the dose of drugs reaching the tumor site is far lower than that of common therapies. However, even with these combined therapies, the median survival rate of 5 years is only 3%–5% (Mojarad-Jabali et al., 2021). Therefore, in order to overcome these limitations, it is urgent to develop a drug delivery system to improve the ability of anticancer drugs to overcome BBB and BBTB and reach tumor cells specifically.

In the past few years, NPs drug delivery systems have been proved that they can enhance the ability of cancer chemotherapy (Lin et al., 2016). Although these drug delivery systems are very promising, they still have some limitations, such as poor cell targeting, premature drug release and lack of real-time monitoring. However, aptamers have the characteristics of strong targeting, easy synthesis and modification, high stability and low immunogenicity, and have great application value in targeted drug delivery. Therefore, after combining NPs with aptamers, NPs can cross the blood-brain/blood tumor barrier, reach glioma cells completely in a long time, and release drugs to achieve effective antitumor treatment (Zhou et al., 2018). At present, a large number of aptamers targeting GBM cell membrane proteins (such as Gint4. T, CL4, AS1411, NOX-A12, GMT8) have been produced, and some of them can cross the blood-brain barrier, and serve as effective therapeutic drugs for GBM animal models. These have been detailed in detail by Cesarini et al. However, although there are several aptamers suitable for targeted drug therapy of GBM, only NOX-A12 aptamer has been recruited for clinical trials (Cesarini et al., 2020).

EGFR is the most over-expressed tyrosine kinase receptor oncogene in all human malignant tumors. It can be activated by combining various growth factors, thus initiating the cascade reaction of signal transduction, promoting cell migration, adhesion, invasion, cell proliferation, angiogenesis and antiapoptosis. EGFR is also the most common oncogene in GBM. The anti-EGFR aptamer selected by Wan et al. It can capture human and mouse GBM cells expressing endogenous wild-type EGFR and mutant EGFRvIII with high sensitivity and specificity (Wan et al., 2010). AuNPs is one of the most widely used high-capacity nanomaterials. It can absorb a large number of photons, increase the local radiation dose in cells, and then damage DNA directly or indirectly, and induce apoptosis, thus showing the ability of radiation sensitization. On the basis of the above experiments, Peng et al. coupled the selected aptamers sensitive and specific to U87-EGFRvIII cells to the surface of AUNP, and then constructed a new brain targeting complex (U2-AuNP), and then carried out experiments *in vitro* and *in vivo* (Peng et al., 2020). The results showed that U2-AuNP could inhibit the proliferation and invasion of U87-EGFRvIII cell line, prevent DNA damage and repair in GBM cells, and prolong the survival time of GBM tumor-bearing mice.

Nanoparticles can not only be used as therapeutic agents, but also be loaded with various antitumor drugs for targeted therapy. For example, GMT8 aptamer (U87 cell specificity) combined with ApNP loaded with docetaxel can significantly induce cell apoptosis and inhibit the growth of tumor cells. *In vivo* imaging in mice showed that ApNP can target glioblastoma and aggregate in tumor site (Gao et al., 2012). The experiment proved that GMT eight aptamer functionalized nanoparticles enhanced the penetration of tumor, improved the targeted therapeutic ability of glioblastoma, and had great potential application value for the treatment and prognosis of brain glioblastoma. In addition, it was found that organic nanoparticle tetrahedral skeleton nucleic acid (tFNA) enhanced the mortality of glioblastoma by activating apoptosis and autophagy. Fu et al. combined tFNA carrying temozolomide (TMZ) with GS24 aptamer, which can specifically bind to transferrin receptor (TRF) of mouse cerebral vascular endothelial cells so that tFNA-TMZ nanoparticles can cross the blood-brain barrier (Fu et al., 2019) (Figures 1B–E). The results showed that tFNA, as a nanocarrier, has a good effect of delivering TMZ, and can enhance the therapeutic effect of glioblastoma.

### Oral cancer

Head and neck cancer is the sixth leading cancer in the world and a major public health problem in India and Southeast Asia (Siegel et al., 2011; Hong et al., 2022). In India, head and neck cancer ranks first among males and third among females (Parkin et al., 2005). Oral cancer (OC) is the most common head and neck cancer, affecting the tongue, lips, alveolar mucosa, floor of the mouth, buccal mucosa, gums, and palate (Kademani et al., 2008). The incidence of OC varies greatly around the world, accounting for 50%–70% of all cancer-related deaths in India and only 5% of malignant tumors in the United States, Western Europe, and Australia. OC is more common in men, and the risk of oral cancer in men is 2–6 times higher than that in women (Sachdeva et al., 2022). Oral squamous cell carcinoma (OSCC) accounts for 90% of OC cases, and it is one of the most challenging diseases, because it tends to metastasize to regional lymph nodes, invade local tissues, and become resistant to chemotherapy drugs. These factors lead to unpredictable prognoses and unfavorable results. About 90% of the failure of radiotherapy and postoperative treatment are due to local recurrence (Yuen et al., 1997; Scully and Bagan, 2007). Although significant progress has been made in treatment methods, including surgical techniques and adjuvant therapy, the general prognosis of OSCC has not improved, which indicates that a new treatment method for OSCC is needed urgently (Lin et al., 2012).

Currently, there have been studies on the active targeted delivery of drugs to oral cancer by aptamer-nano drug delivery system. For example, Mariteset al. Compared the binding affinity and selective targeting of aptamers targeting epidermal growth factor receptor (EGFR) and antibody-coated hollow gold nanospheres (HAuNS) (Melancon et al., 2014). EGFR is overexpressed in 90% of head and neck cancers (Mendelsohn and Baselga, 2003), and the average level of epidermal growth factor receptor between head and neck squamous cell carcinoma and normal tissues is over 13 times (Bonner et al., 2002). By connecting sulfhydryl single-stranded DNA to HAuNS, and then adding complementary RNA targeting EGFR to HAuNS, and combining EGFR targeting aptamer with HAuNS, the multifunctional targeted nano drug apt-HAuNS was obtained. Then, the pharmacokinetics, biological distribution andμSPECT/CT imaging of 111 In labeled apt-HAuNS coupled with anti-EGFR antibody (C225) were evaluated in nude mice with highly malignant human OSC-19 oral tumor. Cell binding *in vitro* and biological distribution *in vivo* showed that apt-HAuNS selectively bound EGFR. μSPECT/CT imaging confirmed that apt-HAuNS had more tumor uptake than C225-HAuNS. The experimental results show that the aptamer targeting EGFR is a promising ligand for targeted delivery of HAuNS, which is used for selective thermal ablation of head and neck cancer with over-expression of EGFR.

Nano-hydroxyapatite (nHAp) has been widely used in the development of imaging probes and drug carriers due to its excellent biological activity and biocompatibility (Oliveira et al., 2019; Marycz et al., 2020). Wenqing Zhang et al. successfully synthesized co-doped nFAp with AS1411 as template by one-pot method, and realized dual-model imaging of AS1411 targeted fluorescence/MRI (Zhang et al., 2021a). AS-nFAp:Gd/Tb has good monodispersity and excellent fluorescence/MRI imaging performance due to the specific binding between AS1411 and upregulated nucleoli in cancer cells. In addition, DOX, a chemotherapeutic drug, was loaded on AS-nFAp:Gd/Tb, and a multifunctional nano probe integrating diagnosis and treatment was constructed. The experimental results *in vitro* confirmed that DOX@AS-nFAp:Gd/Tb had good drug loading capacity and effective pH-induced drug release capacity. *In vivo* anti-tumor experiments show that DOX@AS-nFAp:Gd/Tb has a good anti-tumor effect, and there is no obvious side effect on the mouse model of oral squamous cell carcinoma during the treatment. Generally speaking, DOX@AS-nFAp:Gd/Tb prepared by bionic strategy shows outstanding ability in the identification and treatment of oral squamous cell carcinoma, and has potential clinical application value in the treatment of oral cancer.

### Lung cancer

Lung cancer is the most common cancer in the world, among which non-small cell lung cancer (NSCLC) accounts for about 85% of lung cancer, and is the most common type of lung cancer in the world (Wieleba et al., 2020). NSCLC mainly includes adenocarcinoma, squamous cell carcinoma and large cell carcinoma (Alipoor et al., 2018). For patients with stage I or stage II NSCLC, surgery is the main treatment, and chemotherapy is the auxiliary treatment (Yuan et al., 2019). Patients with advanced NSCLC (stage III/IV) are usually treated with radiotherapy/chemotherapy, immunotherapy or combination therapy (Langer et al., 2013). Although drugs can play a role in the initial stage, cancer cells will develop drug resistance, and drugs have different degrees of toxic and side effects. In 2018, the World Health Organization counted 2.09 million cases of non-small cell lung cancer, and 1.76 million related deaths. Although some progress have been made in the treatment of patients with non-small cell lung cancer, the overall survival rate is still low (Rotoli et al., 2020). Among them, the median total survival time of patients with advanced NSCLC (stage III/IV) is about 10–12 months (Langer et al., 2013). Therefore, the urgent need to develop a high efficiency, specific and low toxicity cancer cell therapy platform is a problem to be solved.

AFNs have great potential application value in the therapeutics of lung cancer. Aptamers functionalized nanomaterials can reduce the immunogenicity of nanoparticles, improve the specificity, prolong the half-life of aptamers in plasma, and improve the stability of AFNs *in vivo* (Wieleba et al., 2020). Zhao et al. isolated aptamer ssDNA that specifically recognizes lung adenocarcinoma for the first time, which provided a foundation for aptamer-targeted therapy of lung cancer (Zhao et al., 2009). At present, AS1411 is the most widely used aptamers for targeted therapy of lung cancer, in addition to MUC1, RA16, GL21. T, S15, anti-EGFR, and so on. Rotoli et al. have made a comprehensive summary on aptamer-targeted drug delivery system (Zhu et al., 2021), so we will not repeat them here. In this article, we mainly introduce the latest experimental research of aptamers nanomaterials in therapeutics of lung cancer.

**FIGURE 2:**
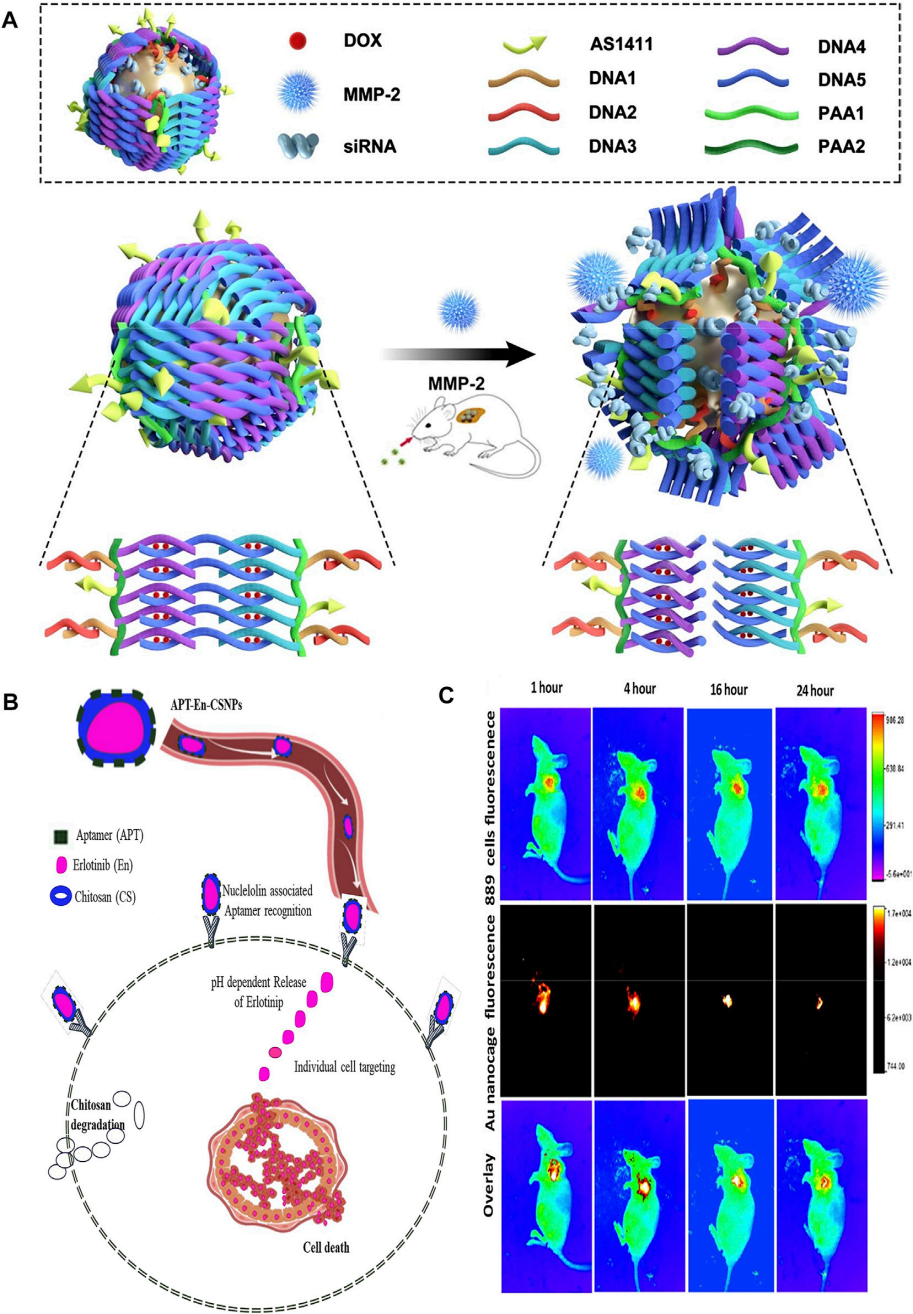
**(A)** The schematic illustration of the construction of gold nanocages and the tumor-induced gene- and DOX-releasing mechanism. **(B)** A schematic demonstration of nuclelolin targeted synthetical route of APT-En-CSNPs, proposed intracellular pH dependent release, penetration and circulation. **(C)** The ability of gold nanocages to deliver anti-VEGF siRNA to non-invasive imagings of NCI-H889 tumor-bearing nude mice *in vivo*. Panel **(B)** was reproduced with permission from Ref. (Saravanakumar et al., 2020), Copyright 2020, Elsevier. **(A,C)** was reproduced with permission from Ref. (Yang et al., 2021), Copyright 2021, Springer Nature.).

Although erlotinib (En) can effectively treat NSCLC, the oral administration of this drug has obvious side effects (such as toxicity, diarrhea, gastrointestinal perforation, and drug resistance) (Spaans and Goss, 2014). Intravenous administration of En is superior to oral administration, so it is very important to choose a tumor specific drug delivery system (DDS) for targeted drug release. Saravanakumar et al. synthesized DDS with double stimulation response, which was used to deliver En at specific sites in cancer microenvironment. They prepared drug-loaded chitosan nanoparticles (APT-En-CSNPs) by loading the drug En with pH-dependent chitosan nanoparticles (CSNPS) and modifying them with aptamer AS1411 (APT). Through pH sensitivity and targeted nucleolar receptor, they released En, and induced apoptosis of A549 cells by producing excessive ROS, nuclear damage and loss ofΔψm (Saravanakumar et al., 2020). The experiment proved that dual stimulation (pH and receptor) responsive drug delivery system can improved the therapeutic effect of En on NSCLC without damaging normal cells (Figure 2B).

It is still a challenge to use combined chemotherapy drugs to enhance the anti-tumor effect while reducing the toxic and side effects of combined drugs. Therefore, Shahriari et al. synthesized a novel nanopolymer based on hyaluronic acid-polycaprolactone by “click chemistry” method, and then coated hydrophilic DOX and hydrophobic camptothecin (CPT) in its inner water chamber and double layer respectively, and the FOXM1 DNA aptamer was bound on the surface of hyaluronic acid shell, thus preparing the combined drug delivery system (Apt-Co-NPs) (Shahriari et al., 2021). Studies have showed that the combination of two anticancer drugs can provide a synergistic effects when FOXM1 aptamer makes cancer cells sensitive to chemotherapy drugs, thus inducing apoptosis. In addition, *in vivo* experiments on SK-MES-1 nude mice showed that the tumor inhibition rates of Apt-Co-NPs and Co-NPs were 87.4% and 74.77%, respectively, which emphasized the sensitizing effect of FOXM1 DNA aptamer on non-small cell lung cancer. These results indicated that the newly developed combined drug delivery platform provides a new way for the synergistic treatment of non-small cell lung cancer with multiple chemotherapeutic drugs.

Gene chemotherapy has become one of the research hotpot in molecular medicine. However, there are still many challenges in developing an efficient, targeted and safe drug delivery system to avoid siRNA degradation and reduce the toxicity and adverse reactions of chemotherapy drugs. Yang et al. proposed an efficient gold nanocage vector (DOX-loaded Au-siRNA-PAA-AS1411) modified by AS1411 aptamer, double-stranded DNA and MMP-2 cleavable polypeptide and loaded with DOX and siRNAs (Yang et al., 2021) (Figure 2A). Among them, gold nanoparticles can be used as X-ray contrast agents to show the release of targeted drugs. The results showed that targeted gene silencing and tumor inhibition were achieved by combined therapy, in which the tumor inhibition rate (tumor signal) of the treatment group was more than 90% and the survival rate was about 67%, while that of the passive gene therapy group was 30% and the survival rate was 0%, which proved that the vector could combine gene therapy, chemotherapy and photothermal therapy (Figure 2C). In addition, this study was the first attempt to construct siRNA nanocarriers, and realize controlled release of siRNA and drugs by using matrix metalloproteinase-2 cleavable polypeptide. Any siRNA used to inhibit lung cancer can be loaded, and the siRNA sequence can also be replaced by other more effective siRNA sequences use for lung cancer or other diseases, showing great clinical application potential. Furthermore, Xu et al. constructed a lipid aptamer nanoplatform (UCILA) loaded with UCNPs and IR-1048 dyes. As mentioned above, not only can five-mode imaging be performed, but also temperature feedback PTT and tumor targeted immunotherapy can be performed simultaneously, which showed excellent ability in real-time monitoring and guiding tumor therapy (Xu et al., 2021).

### Breast cancer

The incidence of breast cancer accounts for 24.2% of all female cancers in the world, and 52.9% in developing countries (Zhu et al., 2021), which is the second leading cause of female death in the world at present (Şahin et al., 2020). Up to now, the treatment of breast cancer mainly depends on systemic and local treatment. Early patients are more likely to choose surgery, while patients with advanced cancer are advised to receive surgery, chemotherapy or radiotherapy. However, chemotherapy drugs have serious side effects, and lead to drug resistance of tumor cells (Liu et al., 2017). Radiation therapy can not only kill tumor cells, but also damage normal tissues, and cannot kill breast cancer cells sufficiently. Therefore, it is necessary to continuously develop new and effective breast cancer treatment methods. The new treatment method of nanomaterials combined with aptamers will contribute to the development of breast cancer therapeutics (Liu et al., 2017; Hu et al., 2019). Aptamers functionalized nanomaterials have the advantages of firm structure, high drug encapsulation efficiency, modifiable release and protection of aptamer from nuclease degradation (Sheikh et al., 2022). They are promising candidate drugs for targeted therapeutics of breast cancer.

**FIGURE 3:**
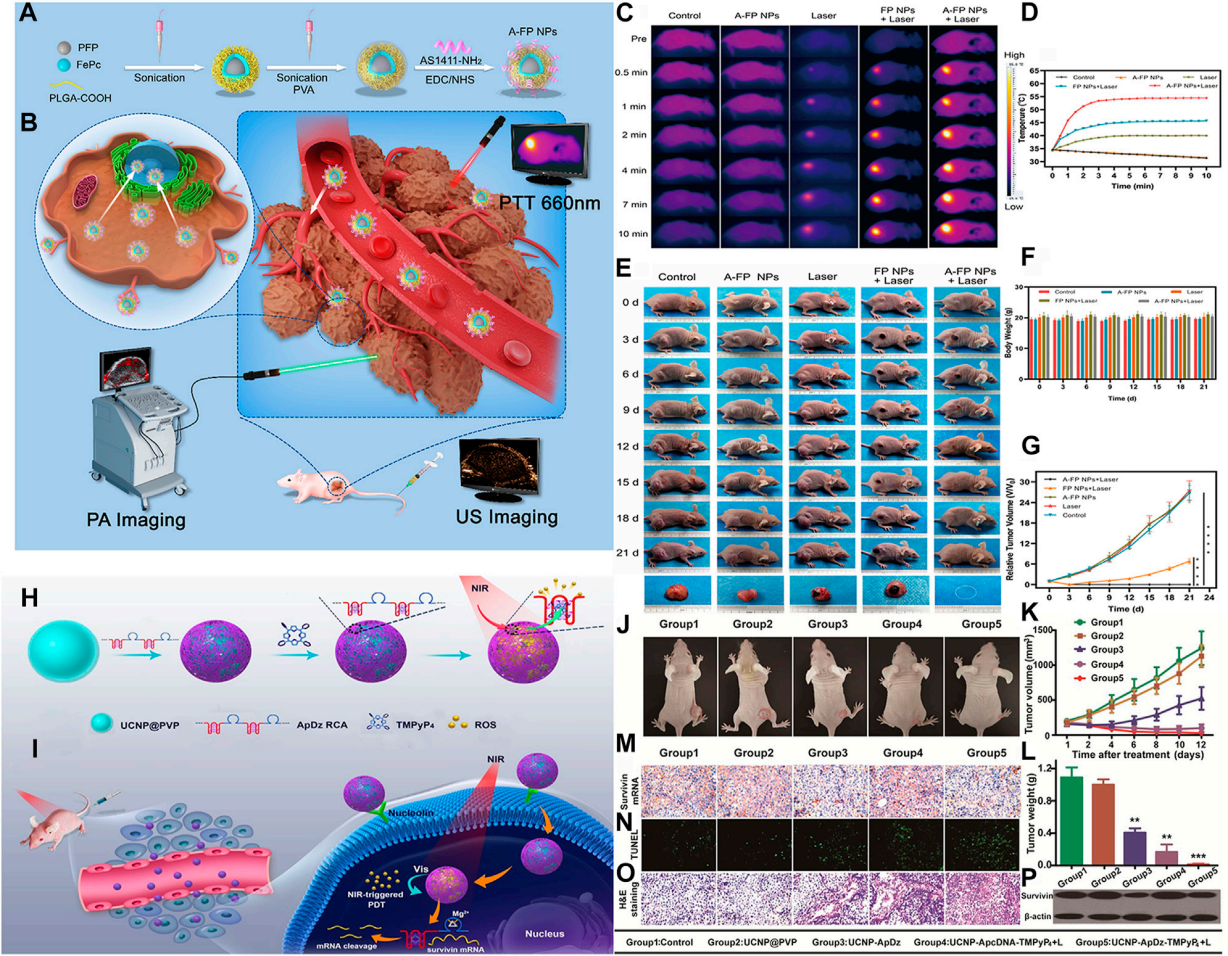
**(A)** Schematic synthetic procedure illustration of synthetic procedure of the multifunctional nanocomplex (A-FP NPs). **(B)** PA/USimaging-guided PTT for breast tumor cells/tissue. **(C)** IR thermal images of MCF-7 tumor-bearing mice with various groups. **(D)** The temperature time curves of tumor. **(E)** The changes in MCF-7 tumor-bearing mice. **(F)** The body weight curves. **(G)** Relative tumor growth curves. **(H)** the synthesis of the multifunctional DNA Polymer assisted upconversion therapeutic nanoplatform. **(I)** The targeted photodynamic nanoplatform for highly efficient photodynamic therapy. **(J)** Photographs of the treated tumor-bearing mice 12 days postinjection. **(K)** Tumor growth curve. **(L)** Tumour weight. **(M)** The mRNA expression of survivin and **(N)** apoptosis of tumors from different groups at 48 h after the indicated treatment. **(O)** histological analysis. **(P)** Western blot analysis. (Panel **(A–G)** was reproduced with permission from Ref. (He et al., 2020), Copyright 2020, DMP. Panel **(H–P)** was reproduced with permission from Ref. (Jin et al., 2020), Copyright 2020, American Chemical Society).

Multifunctional nanoplatform with diagnostic and therapeutic functions has attracted extensive attention in the field of diagnostics and therapeutics. However, some existing nano-drugs have some disadvantages, such as complex preparation process and low specificity. Therefore, there is an urgent need for a multifunctional nanomaterial with strong specificity, high biocompatibility and remarkable therapeutic effect, so as to achieve the integration of diagnosis and treatment and clinical application. He et al. prepared multifunctional nanoparticles (A-FP NPs) (He et al., 2020) with liquid perfluoropentane (PFP) as the core, iron (II)-phthalocyanine (FePc) as the shell, PLGA as the carrier and aptamer AS1411 as the specific targeting component by ultrasonic emulsification (Figures 3A–G). The experimental results *in vitro* showed that A-FP nanoparticles have good stability, biocompatibility and low toxicity, and have active targeting effect on breast cancer cells and tissues. FePc in A-FP NPs have good photothermal conversion ability. Under the irradiation of NIR, even a very low concentration can produce a large amount of heat energy. The experimental results of breast cancer in mice showed that A-FP NPs had significant PTT effects, and there are no obvious recurrence or metastasis in the observation period after tumor resection. In addition, A-FP NPs can also be used as dual-mode contrast agents for PA/US imaging, which can be used for dual-mode omni-directional monitoring of tumors, accurate positioning of tumors and guiding therapeutics. This is a very promising imaging method. The experiment has been proved that A-FP NPs are multifunctional nanoparticles with good biocompatibility, which provide a new idea for clinical transformation of nano-drugs and early diagnostics and therapeutics of breast cancer.

UCNPs is an attractive energy donor because of its characteristic of converting near infrared light into short wavelength photons. In addition, it has the characteristics of non-immunogenicity, good biocompatibility, and no toxic side effects, and is widely used in the field of nanomedicine (Mohan and Poddar, 2021). UCNPs often used as the energy donor of PDT and delivery carrier of photosensitizer, in order to improve the effect of PDT. Jin et al. synthesized a multifunctional DNA polymer up-conversion nanoplatform which can enhance PDT effect. The nanoplatform adsorbed aptamer AS1411 and DNAzyme, which can specifically target tumor cells and carry out gene suppression (Jin et al., 2020) (Figures 3H–P). When nanoplatform was internalized into cancer cells by target, NIR can trigger PDT to produce ROS to kill cancer cells. Moverover, its encoded enzyme can effectively inhibit the expression of Survivin gene, thus improving the efficiency of PDT. *In vitro* and *in vivo* experiments have been proved that multifunctional DNA polymer based on PDT up-conversion nanoplatform has good antitumor effect, which provided a new idea for the application of up-conversion nanoplatform in cancer combined therapy.

The above mentioned experimental studies have reported the applicability of nanodrug delivery system based on PDT in the treatment of breast cancer, but the effect of PDT/biological reducing drug (BD) combined with nanoparticles in the treatment of hypoxic tumors is rarely studied. It has been found that TNBC is more difficult to treat than other types of breast cancer, because there are often areas of low oxygen (Chou et al., 2021). In order to improve the therapeutic effect of tumor, Chou et al. proposed a new therapeutic strategy of PDT/BD for the treatment of TNBC (Chou et al., 2021). They used thin-shell hollow mesoporous Ia3d silica nanoparticles as carrier (MMT-2), loaded with photosensitizer protoporphyrin IX (PPIX) and bioreduction prodrug tirapamine (TPZ), and then combined LXL-1 aptamer with specific recognition ability for TNBC cells with MMT-2 to prepare multifunctional nanoplatform (TPZ @ LXL-1-PPIX-MMT-MMT) *In vitro* experiments showed that TPZ@LXL-1-PPIX-MMT-2 can specifically target TNBC tumor cells. Under the irradiation of laser, PPIX reacted in tumor site to consume O_2_, and then produced ROS with cytotoxicity. The hypoxic tumor microenvironment activated TPZ into toxic free radicals, which can not only removed tumor cells, but also improved the curative effect of PDT. The experiment showed that TPZ@LXL-1-PPIX-MMT-2 nanoparticles can specifically target TNBC tumor cells, effectively killed tumor cells in hypoxic areas, significantly reduced the tumor volume of xenograft mice, prolonged their life cycle, and provided a new therapeutic strategy for TNBC.

### Liver cancer

Liver cancer is one of the most common malignant tumors in the world, and its mortality rate ranks third among all malignant tumors (Hong et al., 2022). There are about 841,000 new cases and 782,000 deaths every year (Zhang et al., 2021b). According to the relevant forecast of the World Health Organization, the number of deaths due to liver cancer will reach 1 million in 2030 (Zhu et al., 2021). HCC is a kind of disease which is difficult to detect in the early stage and develops rapidly. Chemotherapy is still the most common treatment for advanced HCC. However, currently available chemotherapy drugs are mainly passively transported to tumor tissues, lacking specificity and targeting, and have many toxic and side effects, which greatly affect the therapeutic effect (Mohamed et al., 2017; Chen et al., 2019). Therefore, specific delivery of chemotherapeutic drugs to liver cancer cells can effectively control disease progression and overcome the cytotoxic effect of chemotherapeutic drugs on normal cells, which is the main research direction of current liver cancer chemotherapy (Galun et al., 2017; Mohamed et al., 2017). At present, many *in vitro* and *in vivo* experiments have been carried out on the study of active drug delivery to liver cancer cells by aptamer-nanodrug delivery systems (Table 1), aiming at exploring the best drug delivery systems for liver cancer therapeutics and realizing the fundamental and effective therapeutics of liver cancer (He et al., 2021).

**TABLE 1:** Application of aptamers functionalized nanomaterials in the treatment of liver cancer.

Aptamer	Nanocarrier	Therapeutic Agents	Target Cell Line	Therapy	*In Vitro*/*In Vivo*	Model	Ref
Unknown	CNTs	Durvalumab	HepG2 cells	Increase the proportion of T cells and CD8^+^T cells, promote the apoptosis of HepG2 cells.	*In Vitro*/*In Vivo*	Tumor-bearing mouse model	Qiang et al. (2022)
AS1411	CDs	—	Unknown	Silence the expression of FMRP, inhibited the migration and invasive propensity of HCC cells.	*In Vitro*	—	Zhao et al. (2022)
AS1411	Micelles	DOX/miR-519c	HepG2 cells	Inhibits tumor growth, Reverse MDR.	*In Vitro*/*In Vivo*	Mouse model of HCC	Liang et al. (2021)
AS1411	Gold nanoclusters-conjugated chitosan	Methotrexate (MTX)	HepG2 cells	Significantly release MTX in intracellular lysosome of tumor cells.	*In Vitro*	—	Zhang X. et al. (2021)
A54/A15	Liposomes	Salinomycin/oxaliplatin	BEL-7402 cells	Kill tumor cells expose CD133+ cancer cells.	*In Vitro*	—	Jianghong et al. (2021)
EpCAM	Silica NPs	Sorafenib and CRISPR/Cas9	H22 cells	Achieved efficient EGFR gene therapy, caused tumor inhibition.	*In Vitro/In Vivo*	Kunming mice models	Zhang et al. (2020)
EpCAM	RNA nanospheres	Sorafenib/siRNA	—	Target delivery of suitable drugs for treatment.	*In Vitro/In Vivo*	—	Chen et al. (2020)
EpCAM	UA/polyphenol (EGCG)	—	HepG2 /HeLa cells	Activate innate immunity and acquired immunity to produce synergistic therapeutic effect.	*In Vitro*/*In Vivo*	Male KM mice model	Zhang B. et al. (2021)
TLS 9a	phosphorothioate backbone	Paclitaxel	HepG2 /Huh-7 cells	Targeting specific neoplastic hepatocytes, Leading to apoptosis of tumor cells.	*In Vitro*/*In Vivo*	Male SD rats model	Chakraborty et al. (2020b)
TLS 9a	Phosphorothioate backbone	Paclitaxel	HepG2 /Huh-7 cells	Inducing selective apoptosis of tumor hepatocytes.	*In Vitro*/*In Vivo*	SD male rats model	Chakraborty et al. (2020a)
TLS11a	GQDs/magnetic chitosan	Adriamycin	H22 cells	Inhibit tumor growth , extend the survival duration of tumor-bearing mice.	*In Vitro*/*In Vivo*	Female H-2Kd BALB/c nude mice model	Chen et al. (2022)
TLS11a	Silica NPs/liposomes	DOX	H22 cells	Target liver cancer tissue and deliver DOX to the nuclei of liver cancer cells.	*In Vitro*/*In Vivo*	BALB/c Mice model	Ding et al. (2020)
TLS11a	BPQDs	—	HepG2 cells	Act as an active targeting nanocatalyst for programmable killing of cancer cells in hypoxic TME.	*In Vitro*/*In Vivo*	Mouse model of HCC	Lan et al. (2019)
TLS11a	Anti-CD3	—	H22 cells	Guide T cells to kill tumor cells, enhance the anti-tumor effect.	*In Vitro*/*In Vivo*	Female BALB/c mice model	Hu et al. (2018)
TLS11a	GNP	—	HepG2 cells	Significantly enhanced chemotherapy efficiency.	*In Vitro*/*In Vivo*	HepG2-bearing nude mice model	Zhang D. et al. (2017)
ST21	PEG	MiRNA-195 /fasudil	SK-Hep-1 cells	Had strong silencing activity of ROCK2 and VEGF.	*In Vitro*/*In Vivo*	Nude mice model of HCC	Liu et al. (2016)

**FIGURE 4:**
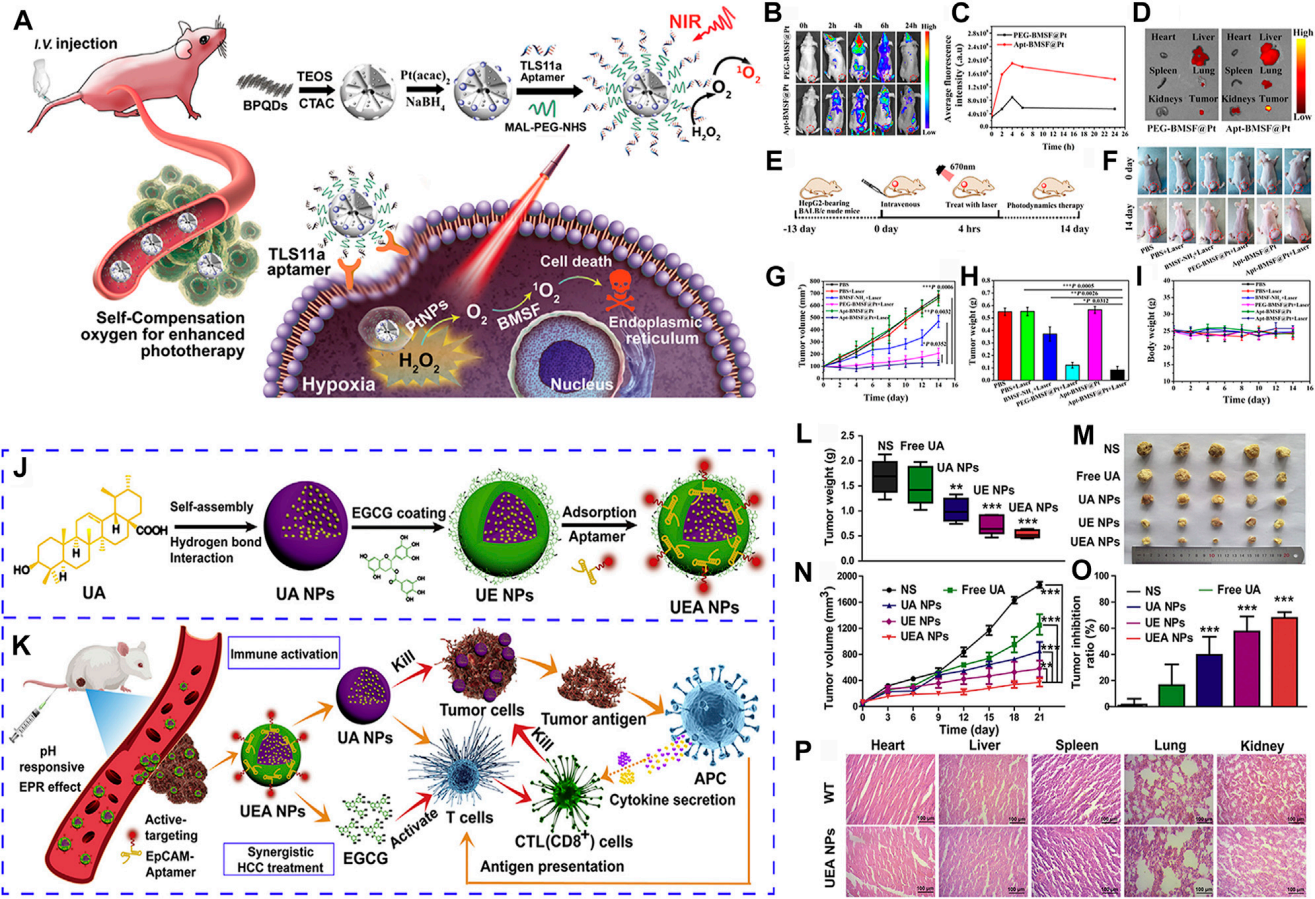
**(A)** Schematic illustration of the self-regulation of precise cancer phototherapy in vivoa. **(B)** *In vivo* fluorescence imaging. **(C)** Quantification of the fluorescence intensity of tumors. **(D)** *Ex vivo* fluorescence images of tumor and organs. **(E)** Schematic illustration of a typical therapeutic procedure. **(F)** *In vivo* response to PBS, BMSF-NH2, PEG-BMSF@Pt, and Apt-BMSF@Pt with orwithout laser irradiation. **(G)** Tumor volume change. **(H)** Average tumor weight. **(I)** Mean body weight. **(J)** The preparation of the “carrier-free” Apt-modified nanodrug based on the UA and EGCG. **(K)** Synergistic HCC treatment of the nanosystem by activating the innate and acquired immunity. **(L)** Tumor weight excised from mice. **(M)** Image of tumor tissues separated from mice treated with different formulations. **(N)** Tumor volume growth curves. **(O)** Tumor inhibition ratio. **(P)** HE staining of heart, liver, spleen, lung and kidney from mice of WT and UEA NPs. Panel **(A–I)** was reproduced with permission from Ref. (Lan et al., 2019), Copyright 2019, American Chemical Society. Panel **(J–P)** was reproduced with permission from Ref. (Zhang et al., 2021b), Copyright 2021, Elsevier.).

Black phosphorus quantum dots (BPQDs) have unique photocatalytic activity, but the low oxygen tumor microenvironment (TME) seriously hinders their biological application. Recently, Lan et al. constructed the TLS11a aptamer/Mal-PEG-NHS modified BPQDs/platinum hybrid mesoporous silica skeleton nanosystem (Apt-BMSF@Pt), which have the advantages of good environmental stability, active targeting to liver cancer cells and self-supply of oxygen under H_2_O_2_ conditions (Lan et al., 2019). The experimental results showed that Apt-BMSF@Pt can significantly improved the efficiency of TME *in vitro* and *in vivo* (Figures 4A–I). *In vivo* fluorescence imaging of mouse liver cancer showed that Apt-BMSF@Pt could effectively accumulate in liver cancer cells, and improved TME induced by hypoxia through self-compensation mechanism. Therefore, APT-BMSF@Pt can be used as an active targeting nanocatalyst for accurate cancer treatment in self-regulating hypoxic TME.

For the first time, Chakraborty et al. used aptamer TLS 9a (L5) with sulfur skeleton modification for drug delivery of liver cancer cells, and loaded PTX to construct L5 functionalized drug nanocarrier (PTX-NPL5) (Chakraborty et al., 2020a). Then, the therapeutic effects of PTX-NPL5 were compared with other drug nanocarriers functionalized with aptamers and non-aptamers (such as galactosamine and apolipoprotein) reported previously. The experimental results showed that the interaction between L5 and TAG-72/HSP70 leaded to the preferential accumulation of PTX-NPL5 in hepatoma cells, and L5 has showed greater advantages than previous targeting ligands for hepatoma cells. The interaction between L5 and the surface biomarker protein uniquely expressed by tumor liver cells provided great hope for developing effective precision drugs in the near future to fundamentally improved the survival of liver cancer patients.

At present, most nanocarriers have some disadvantages, such as potential toxicity and unclear metabolism *in vivo*, which have some limitations in clinical application. Natural ursolic acid (UA) and polyphenol (EGCG) have good anti-cancer activity and low toxicity, and become potential nano-carriers for *in vivo* administration. Zhang et al. used UA and EGCG as raw materials to construct a novel “core-shell” co-assembled carrier-free nano-system (UEA) through the modification of EpCAM aptamers, which can be used for the synergistic treatment of liver cancer (Zhang et al., 2021b) (Figures 4J–P). It has been found that UEA nanoparticles can activate innate immunity and acquired immunity for immunotherapy, and have significant synergistic therapeutic effects. UEA nanoparticles have the advantages of stronger stability, sensitive pH response, low toxicity, strong permeability to tumor tissues and strong tumor accumulation ability, which will provide a new idea for the future research and development of self-assembled drug delivery systems and an effective intervention strategy for therapeutics of clinical liver cancer.

### Pancreatic cancer

Pancreatic cancer is one of the most serious malignant tumors, and it is also called the king of cancer (Tian et al., 2021). Its incidence rate ranks ninth, and its 5-year survival rate is <1% (Bray et al., 2018). It is predicted that by 2030, pancreatic cancer will surpass breast cancer, prostate cancer and colorectal cancer, and become the second leading cause of cancer-related death (Hong et al., 2022). At present, the conventional treatment strategies for pancreatic cancer include surgery, chemotherapy and radiotherapy. However, due to the difficulty of early diagnosis, the 5-year survival rate is low (Izumi et al., 2019), and the therapeutics effect is almost unsatisfactory. In the process of chemotherapy, small molecule drugs entering the body through the blood circulation, and more than 90% of them are absorbed by normal tissues, and only 2%–5% of them can reach the tumor site (Tian et al., 2021). Therefore, there is urgent need to actively study and develop a new effective strategy to improve the prognosis of pancreatic cancer. At present, a variety of aptamers modified nanomaterials have been developed for targeted drug delivery of pancreatic cancer (Table 2), but all of them are limited to *in vitro* experiments or *in vivo* experiments of animals. There is no aptamer-nanodrug delivery systems for clinical therapeutics of pancreatic cancer patients.

**TABLE 2:** Application of aptamer functionalized nanomaterials in the treatment of pancreatic cancer.

Aptamer	Nanocarrier	Therapeutic Agents	Target Cell Line	Therapy	*In Vitro*/*In Vivo*	Model	Ref
AS1411	Atelocollagen	Gemcitabine	Pancreatic cancer PDX cells	Target delivery of anticancer drugs, enhance tumor margin negative resection	*In Vitro/In Vivo*	Pancreatic cancer PDX mice model	Hong et al. (2022)
AS1411	GNR	—	PANC-1/HT-1080 cells	*In vitro* efficacy of aptamer-loaded AuNS can be enhanced by increasing the loading of G-quadruplex homodimer AS1411 on the surface of AuNS.	*In Vitro*	—	Dam et al. (2014)
AS1411	PEG-PDLLA	Triptolide (TP)	MIA PaCa-2 cells	Accumulate in tumor tissues and target CPC cells, Prolong the life of tumor mice	*In Vitro/In Vivo*	MIA PaCa-2 cell-bearing mice model	Wang et al. (2016)
XQ-2d	Polymeric micelles	DOX	Panc-1 cells	Target delivery of anticancer drugs penetrated into 3D spheres of Panc-1 cells	*In Vitro*	—	Tian et al. (2021)
XQ-2d	Diacyl phospholipid	—	PL45 cells	Lipid-DNA-aptamer-modified T-lymphocytes bound to PL45 cells and kill PDAC	*In Vitro*	—	Zhong et al. (2020)
J10	Lipid nanovectors	Gemcitabine/ IOX2	KPC cells	Target to the surface of monocytes, adapt to treat a myriad of diseases that involve monocyte recruitment	*In Vitro/In Vivo*	Orthotopic PDAC mouse model and its liver metastasis model/Myocardial IR injury model	Huang et al. (2021)
Ap52	Phosphorothioate	DOX	AsPC-1/Cal-27/MCF-7/SK-MEL-28/OMF cells	Selectively enter cancer cells, enhance the toxicity to cancer cells	*In Vitro/In Vivo*	Pancreatic cancer xenograft male nude mice model	Wang et al. (2021)
GBI-10	Cell-penetrating peptide(CPP)	Camptothecin prodrug(CPTD)	Luc-iMiapaca cells	Target drug delivery and obvious anti-tumor effect	*In Vitro/In Vivo*	Miapaca orthodox pancreatic cancer xenograft mice model	He et al. (2018)
AP1153	Fluorescent NPs	—	PANC-1 cells	Target entry into tumor cells and inhibit PDAC cell proliferation.	*In Vitro/In Vivo*	Orthotopic PDAC xenografts athymic male nude (nu/nu) mice model	Clawson et al. (2017)
Nnknow	PLGA	Ormeloxifene	HPAF-II/AsPC-1/BxPC-3/Panc-1/MiaPaca	Target tumor cells and inhibit the growth of tumor cells.	*In Vitro/In Vivo*	BxPC-3 xenograft mice model	Khan et al. (2015)
PL8	Nanotrain construction (NT8)	DOX	PL45 cells	Target specific tumor cells and inactivate some tumor cells	*In Vitro*	—	Champanhac et al. (2015)

**FIGURE 5:**
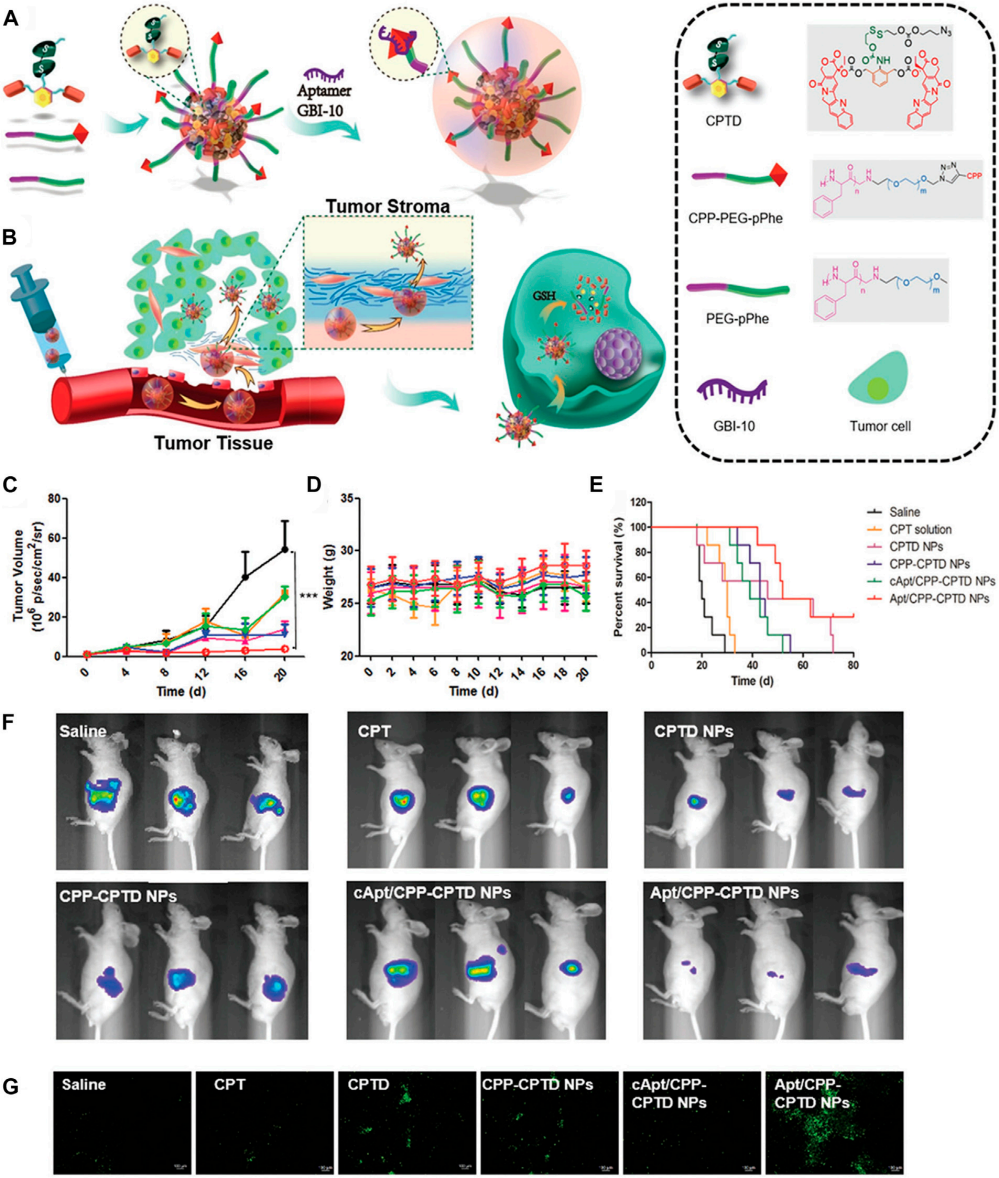
**(A)** Schematic illustration of Apt/CPTD NPs formulation. **(B)** Sequentially triggered tumor stroma permeability of Apt/CPTD NPs and intra-cellular redox-triggered drug release. **(C)** Tumor volume changes and **(D)** body weight on day 0, 3, 6, 9, and 12. **(E)** Survival curves after five doses of administration. **(F)** Bioluminescence images of Miapaca xenograft models on day 12. **(G)** Representative histological images of Miapaca tumor xenografts of treated groups using the TUNEL assay. Green: apoptosis cells. (Reproduced with permission from Ref. He et al. (2018)).

In order to overcome the serious drug resistance of pancreatic vessel element cancer (PDAC) to traditional therapy, He et al. modified cell penetrating peptide (CPP) with extracellular matrix component EMC (tenecin-C) targeting aptamer (GBI-10), which was used to disguise CPP *in vivo* and homing PDAC, and then designed and synthesized a sequential response aptamer/cell penetrating peptide-camptothecin prodrug nanoparticles (Apt/CPP-CPTD NPs) (He et al., 2018). The Apt/CPP-CPTD NPs in PDAC matrix are affected by Tenascin-C, which can separate GBI-10 from CPP. The exposed CPP can promote the further penetration of nanoparticles in tumor tissues and the phagocytosis of tumor cells. After entering PDAC cells, the high redox potential in cells can further trigger the controlled release of chemical drugs. After entering PDAC cells, the high redox potential in cells can further trigger the controlled release of chemical drugs (Figure 5). The results showed that Apt/CPP-CPTD nanoparticles had mild cytotoxicity *in vitro*, good anti-tumor effect *in vivo*, and significantly reduced the systemic toxicity of CPTD. This unique sequence triggered nanoplatform has the functions of tumor penetration and intelligent drug release, and is expected to become a promising PDAC therapy strategy.

Abnormal expression of extracellular matrix in tumor stroma forms a dense physical barrier, which leads to extravasation and insufficient infiltration of nanotherapy (Wang et al., 2022a). Huang et al. modified lipid nanocarriers (LPNs) with J10 aptamer and loaded gemcitabine on LPNs, and synthesized nanoparticles J10-GEM-LNPS with active targeting to tumor cells (Huang et al., 2021). The nanoparticles can attach to monocytes, leave blood vessels, and penetrate the dense extracellular matrix with monocytes, thus improving the efficiency of drug delivery. The results showed that the amount of gemcitabine reaching the tumor site was significantly increased, which could significantly reduce the primary and metastatic foci of PDAC mice, improve the overall survival rate of tumor mice, and had no obvious toxicity to liver and kidney. The lipid nanocarrier is easy to prepare, and is expected to serve as a drug delivery platform for various diseases related to monocyte recruitment. The only drawback of this study was that the researchers only treated the mice for a short time. Although the overall condition and survival rate of the tumor mice were improved, it was not completely cured. Therefore, it is necessary to carry out longer-term research in the future.

### Colorectal cancer

Colorectal cancer (CRC) is the third most common cancer in the world (Jaferian et al., 2016) with a very high mortality rate. In 2020, the American Cancer Society conducted an investigation on the incidence of colorectal cancer. About 147,950 patients have been diagnosed with colorectal cancer, and the number of death was as high as 53,200. The number of patients under the age of 50 who died of colorectal cancer was 3,640 (Zahiri et al., 2021). The onset age of colorectal cancer tends to be younger and younger (Siegel et al., 2020). Surgical resection and chemotherapy are the main treatments for colorectal cancer (Telang, 2022). In order to reduce the side effects of chemotherapy drugs and realize the targeted delivery and release of drugs, people have developed various nanodrug delivery systems modified by aptamers, including polymers, lipid nanoparticles, inorganic nanoparticles, micelles and so on (Ahmadyousefi et al., 2019).

**FIGURE 6:**
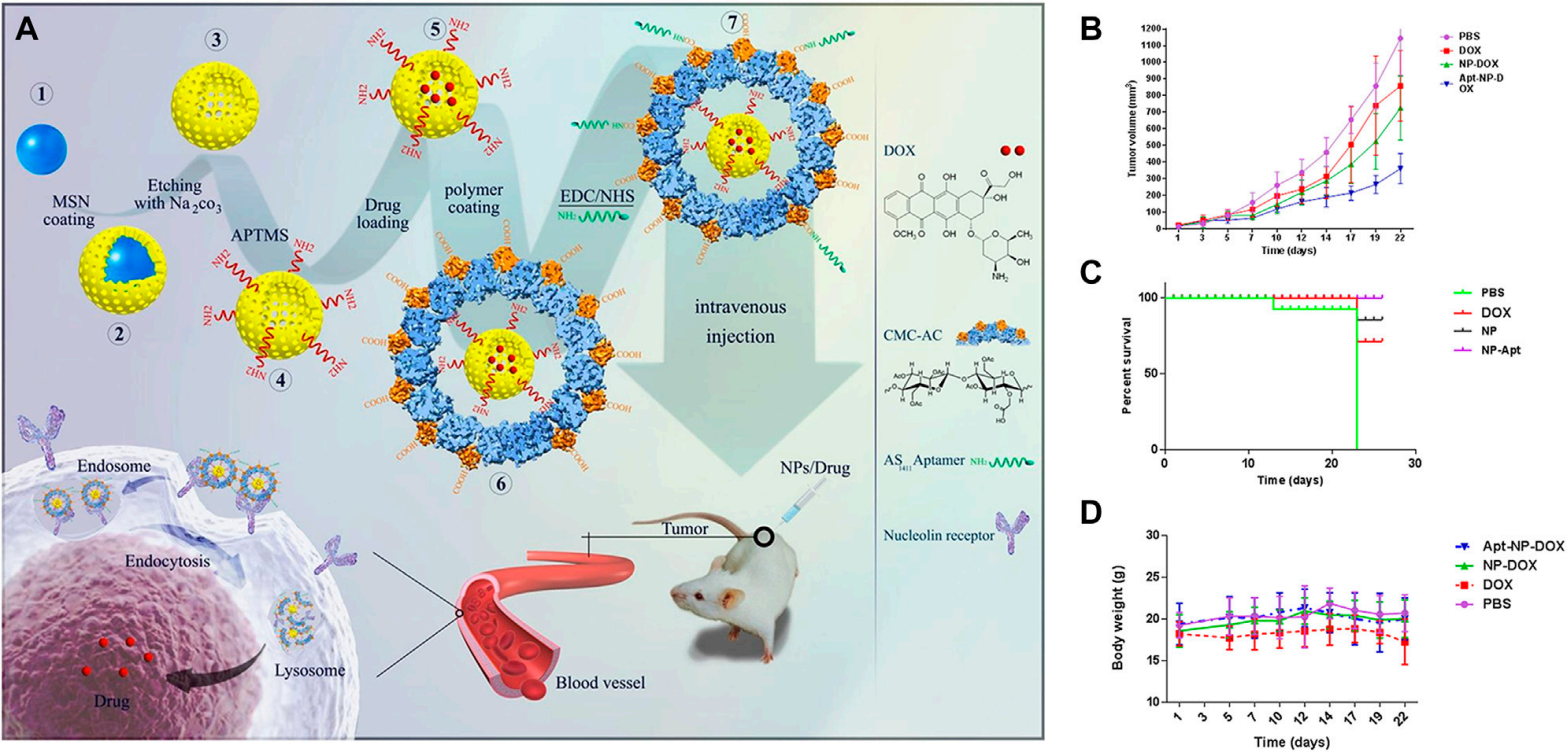
**(A)** Schematic presentation of the preparation of HMSNs, their coating with acetylated CMC and functionalization with AS1411 aptamer for targeting nucleolin overexpressing cancer cells. Tumor size **(B)**, Survival curve **(C)**, and Body weight loss **(D)**. Reproduced with permission from Ref. (Nejabat et al., 2018), Copyright 2018, Elsevier.

Nanodelivery systems modified by aptamesr have been widely studied in the therapeutics of colon cancer, and a large number of *in vitro* and *in vivo* experiments have been carried out. Table 3 lists the related experimental studies in the past 10 years in detail. Several typical or novel drug delivery systems will be introduced in the following. For example, Nejabat et al. coated acetylated carboxymethyl cellulose (Ac-CMC) on the surface of HMSN loaded with DOX, and then covalently linked to AS1411 aptamer to guide drug delivery to cancer cells with high nucleolar expression (Nejabat et al., 2018). *In vitro* studies have been confirmed that AS1411 modified Ac-CMC/HMSN specifically targets MCF-7 and C26 cells, resulting in a significant decrease in tumor cell activity, but no obvious toxicity to normal cells. In C26 tumor-bearing mice, aptamer-coupled nanoparticles showed obvious advantages in inhibiting tumor growth and improving survival rate (Figure 6). It was confirmed that Ac-CMC/HMSN modified by AS1411 has great potential as a new targeting carrier. In another study, researchers loaded Apt-LP3-CPT lipid polymer drug delivery system with APT-LP3-CPT lipid polymer modified by AS1411 aptamer (PEGLA) and dipalmitoyl phosphatidylcholine (DPPC) (Zahiri et al., 2021). *In vitro* and *in vivo* experiments of C26 tumor-bearing mice showed that Apt-LP3-CPT had good stability and high drug loading efficiency, and it could specifically aggregate in tumor cells and inhibit the growth of tumor tissues. The polymer-rich lipid polymer platform designed and synthesized in this study provided a good prospect for the development of effective anticancer nano-drugs.

**TABLE 3:** Application of aptamer functionalized nanomaterials in the treatment of colorectal cancer.

Aptamer	Nanocarrier	Therapeutic Agents	Target Cell Line	Therapy	*In Vitro*/*In Vivo*	Model	Ref
AS1411	PEGylated solid lipid NPs	DOX	C26 cells	Target drug delivery to inhibit tumor growth	*In Vitro/In Vivo*	Bearing the C26 tumor mice model	Shakib et al. (2022)
AS1411	Polycaplactone-poly (glyceryl methacrylate) hybrid polymersomes	DOX	HT29 and C26 cells	Target drug delivery system can be used for both tumor diagnosis and treatment.	*In Vitro/In Vivo*	C26 tumor-bearing BALB/c mice model	Babaei et al. (2022)
AS1411	Poly carboxylic acid dextran coast PLA-PEI micelles	CPT /survivin-shRNA	C26 cells	Multi-functional drug delivery carriers significantly inhibit tumor growth.	*In Vitro/In Vivo*	Female BALB/c mice colon carcinoma model	Sanati et al. (2022)
AS1411	Lipopolymersomes	CPT	HT29 and C26 cells	Targeted drug delivery with high carrying capacity	*In Vitro/In Vivo*	C26 tumor bearing BALB/c female mice model	Zahiri et al. (2021)
AS1411	Albumin NPs	DTX	CT26 cells	Significantly enhance the anti-tumor effect and prolong the survival time of colon cancer mice.	*In Vitro/In Vivo*	BALB/c female mice colon carcinoma model	Yu et al. (2020)
AS1411	DNA nanoparticles	DOX	CT26 cells	Target drug delivery to enhance anti-tumor effect.	*In Vitro/In Vivo*	BALB/c mice colon carcinoma model	Yao et al. (2020)
AS1411	PEGylated rod-shaped mesoporous silica NPs	CPT/iSur-DNA	C26 cells	Improve the pharmacokinetics of targeted drugs, promote cell apoptosis, inhibit tumor growth rate	*In Vitro/In Vivo*	C26 tumor bearing mice model	Babaei et al. (2020)
AS1411	PLGA	DOX	C26/C6 cells	Target drug delivery into cell lines with high nucleolar expression	*In Vitro*	—	Mosafer et al. (2018)
AS1411	Silica NPs	DOX	HCT116 cells	Safe targeted drug delivery system *in vivo*	*In Vitro*	—	Thorat et al. (2020)
AS1411	HMSNs	DOX	MCF-7 and C26 cells	High drug loading, targeted drug delivery, effective inhibition of tumor growth.	*In Vitro/In Vivo*	Bearing C26 cells BALB/c female mice model	Nejabat et al. (2018)
AS1411	PLGA	DOX	C26 cells	Targeted drug delivery, inhibiting tumor growth and prolonging the life cycle of colon cancer mice.	*In Vitro/In Vivo*	Bearing C26 cells BALB/c mice model	Mosafer et al. (2017)
AS1411	Camptothecin-loaded pegylated PAMAM dendrimer	CPT	C26 cells	Provide site-specific delivery of camptothecin, inhibit C26 tumor growth and significantly decrease systemic toxicity	*In Vitro/In Vivo*	C26 tumor-bearing BALB/C mice model	Alibolandi et al. (2017)
MUC-1	Gold coating of SPIONs	—	HT-29 cell lines	Target colon cancer cells, phototherapy leads to cell death	*In Vitro*	—	Azhdarzadeh et al. (2016)
MUC-1	PEGylated Au dendrimer	CUR	HT29/C26 cells	Target drug delivery to improve the anti-tumor ability of drugs.	*In Vitro/In Vivo*	C26 tumor bearing BALB/c female mice model	Alibolandi et al. (2018)
MUC1	Chitosan NPs	SN38	HT-29 cell lines	Increase the solubility of drugs, deliver drugs to tumor cells specifically, and increase the efficacy of drugs.	*In Vitro*	—	Sayari et al. (2014)
MUC1	DNA micelles hybrid NPs	DOX/pro-apoptotic peptide (KLA)	MCF-7/ C26 cells	Double targeted drug delivery can significantly improve the therapeutic effect of drugs and inhibit tumor growth.	*In Vitro/In Vivo*	C26 tumor bearing mice model	Charbgoo et al. (2018)
MUC1/AS1411	Dendrimer	EPI	MCF7 and C26 cells	Targeted drug delivery to inhibit the growth of tumors *in vivo* and *in vitro*.	*In Vitro/In Vivo*	C26 tumor-bearing BALB/C mice model	Taghdisi et al. (2016)
SYL3C	PEGylated-nanoliposomal	DOX	C26 cells	Target drug delivery improve the aggregation of drugs in tumors and prolongs the survival time of tumors	*In Vitro/In Vivo*	Female BALB/c mice model bearing C26 tumors	Mashreghi et al. (2020)
EpCAM	Carboxyl modified MSNs	DOX	SW620 cells	Target delivery of therapeutic agents into EpCAM positive colon cancer cells to improve therapeutic index while reducing side effects	*In Vitro*	—	Xie et al. (2016)
EpCAM	Star-shaped micelle	CPT	HT29 and C26 cells	Targeted drug delivery can improve the therapeutic effect of drugs, inhibit the growth of tumors and prolong the survival time of tumor mice	*In Vitro/In Vivo*	BALB/c C26 tumor-bearing mice model	Sahranavard et al. (2021)
EpCAM	Lipid-polymer-lecithin hybrid NPs	CUR	HT29 cells	Improve the targeting of chemotherapy drugs to colorectal cancer cells.	*In Vitro*	—	Li et al. (2014)
LA1	PEI-modified grapefruit-derived nanovectors	DOX/siRNA	LoVo/MDR cell lines	Down-regulate the expression of P- glycoprotein, inhibit cell proliferation and promote cancer cell apoptosis.	*In Vitro/In Vivo*	BALB/c and BALB/c SCID mice model	Yan et al. (2018)
FKN-S2	Micelles functionalized with an outer PEG	—	MCA-38.FKN cells	Fractalkine may serve as a specific target for nanoparticle delivery to cancer cells	*In Vitro/In Vivo*	Female homozygous mice model	Harris et al. (2018)
5TR1	PEGylated liposomal	DOX	C26 cells	Target drug delivery, inhibit tumor growth and prolong the survival time of colon cancer mouse model	*In Vitro/In Vivo*	C26 tumour-bearing mice model	Moosavian et al. (2018)
5TR1	SPIONs	EPI	C26 cells	Target drug delivery	*In Vitro/In Vivo*	Mice model of colon carcinoma	Jalalian et al. (2013)
5TR1	Chitosan-modified PLGA	EPI	MCF7 and C26 cells	Target drug delivery can significantly inhibit tumor growth	*In Vitro/In Vivo*	Bearing C26 cells BALB/c mice model	Taghavi et al. (2017)

Qian group research showed that COX-2 can catalyze the oxidation of dihomo-γ-linolenic acid (DGLA) to produce a unique anticancer by-product 8-hydroxy hexanoic acid (8-HOA), which can be used as histone deacetylase inhibitor (HDACi) to inhibit the growth and migration of cancer cells/tumors (Xu et al., 2018a; Xu et al., 2018b; Yang et al., 2019). However, DGLA can be decomposed by D5D, which greatly limited its biomedical application. Furthermore, Xu et al. have proved in previous experiments that DGLA together with Delta-5-desaturase (D5D)siRNA can promote COX-2-catalyzed DGLA peroxidation to form 8-HOA, thus effectively inhibiting the growth of tumor cells. However, due to its “off-target” effect, it was still a huge challenge to deliver naked siRNA. Subsequently, Xu et al. Innovatively for the first time, three WJ RNA nanoparticles modified by EpCAM aptamer were used to specifically deliver D5D-siRNA into human colon cancer HCa-7 cells, which resulted in a significant downregulation of D5D expression, thus inducing a higher DGLA/AA ratio and promoting the formation of 8-HOA at the threshold level (Xu et al., 2019). *In vivo* study of HCa-7 tumor-bearing mice showed that DGLA combined with 3WJ-EpCAM-siRNA could significantly inhibit the growth of tumor. The experiment have confirmed that three WJ-EpCAM nanodrug delivery systems have been developed a promising therapeutics strategy for colon cancer, and it is expected to be applied in clinic in the near future.

### Ovarian cancer

Ovarian cancer is a serious gynecological malignant tumor with a high incidence (Sung et al., 2021), with more than 220,000 cases worldwide every year (Hoosen et al., 2018). According to the latest data of new cancer cases and deaths in 2021 evaluated by American Cancer Society (Siegel et al., 2021), 13,770 out of 21,410 ovarian cancer patients died, accounting for about 5% of all malignant tumors, making ovarian cancer the second most common but deadly malignant tumor of female reproductive system. Surgical resection and chemotherapy are the main treatments for tumor. Although the early treatment is effective, there will be some problems such as recurrence, unbearable side effects and drug resistance after therapeutics of advanced ovarian cancer (Menderes et al., 2017; Jiang et al., 2020). Therefore, new therapeutics strategies should be adopted to improve the therapeutic effect of ovarian cancer.

AFNs (Table 4) can specifically target ovarian cancer cells and release drugs into tumor tissues, but they have no targeting effect on normal tissues. This treatment method has obviously enhances the toxicity of drugs to tumor cells and obviously reduces the side effects of drugs to normal tissues. For example, Savla et al., synthesized a quantum dot-MUC1-doxorubicin complex (QD-muc1-doxorubicin) with tumor targeting and pH response, which was used for chemotherapy of ovarian cancer (Savla et al., 2011). Confocal microscope and *in vivo* imaging studies show that QD-MUC1-DOX can preferentially target multidrug-resistant ovarian cancer cells, effectively release DOX at acidic pH, and its toxicity to ovarian cancer cells is higher than that of free DOX. Ghassami et al. prepared polybutylene adipate-butylene terephthalate (Ecoflex®) nanoparticles (APT-DTX-NPS) modified by HER-2 and loaded with DTX (Ghassami et al., 2018). Compared with DTX-NPs and free drugs, the cytotoxicity and cellular uptake of Apt-DTX-NPs in HER-2 high-expression cell line were significantly enhanced, and the pharmacokinetic parameters of tumor-bearing B6 nude mice with HER-2 high expression *in vivo* were also significantly increased. Subsequently, the team evaluated the blood and tissue toxicity and biodistribution of Apt-DTX-NPs (Ghassami et al., 2019). The results showed that the targeted drug delivery system can effectively concentrate the drugs on the tumor site, and has no obvious toxic and side effects on the surrounding normal tissues. APT-DTX-NPs, as a non-toxic, biodegradable and biocompatible delivery system, has great clinical application potential.

**TABLE 4:** Application of aptamer functionalized nanomaterials in ovarian cancer.

Aptamer	Nanocarrier	Therapeutic Agents	Target Cell Line	Therapy	*In Vitro*/*In Vivo*	Model	Ref
MUC16	MNPs	erlotinib	Ovarian cancer cells	Targeted drug delivery, realizing combined drug delivery under the guidance of images.	*In Vitro/In Vivo*	Ovarian cancer-bearing mice model	Mohaghegh et al. (2022)
MUC1	Carboxyl terminated QDs	DOX	A2780/MDR ovarian cancer cells	Targeted drug delivery can inhibit the growth of multidrug resistant tumor cells.	*In Vitro/In Vivo*	Ovarian cancer xenograft female nude mice model	Savla et al. (2011)
EpCAM	PLGA	—	A2780/ OVCAR-3 cells	Aptamer targeting has obvious specificity for ovarian cancer cells.	*In Vitro*	—	Wu et al. (2021)
AS1411	PLGA-PEG	Cisplatin /anti-miR-21	CIS-resistant A2780 cells	Specific targeted drug delivery, reduce the drug resistance of drug-resistant tumor cells, and enhance the effect of drug therapy on tumors.	*In Vitro*	—	Vandghanooni et al. (2018)
AS1411	Liposome	MicroRNA 29b	A2780 cells	Targeted drug delivery can increase the concentration of therapeutic drugs and improve the therapeutic effect.	*In Vitro*	—	Jiang et al. (2020)
AS1411	star-shaped glucose-core polycaprolactone-PEG	Cisplatin/anti-miR-214	A2780 cells	Targeted drug delivery can reduce drug resistance of tumor cells and enhance cell apoptosis.	*In Vitro*	—	Vandghanooni et al. (2020)
HER-2	Ecoflex® polymeric NPs	Docetaxel (DTX)	SKOV-3 cells	Targeted drug delivery, no toxic or side effects on surrounding organs and tissues.	*In Vitro/In Vivo*	Bearing SKOV-3 cells nude mice model	Ghassami et al. (2019)
HER-2	Ecoflex®polymeric NPs	DTX	SKOV-3/MDA-MB-468 cells	Specific targeted drug delivery, highly enhance the effect of drug therapy on tumor.	*In Vitro/In Vivo*	Tumor induction in female athymic B6 nude mice	Ghassami et al. (2018)
Annexin A2	Phi29 pRNA 3WJ motif	DOX	SK-OV3 cells	Highly specific targeted drug delivery can enhance the effect of drug therapy on tumors.	*In Vitro/In Vivo*	Ovarian cancer xenograft female nude mice model	Pi et al. (2017)
VEGF	Au-FeO NPs	Notch3 siRNA	MDR ovarian cancer cells	could reverse MDR of ovarian cancer cells against the chemotherapeutic drug cisplatin	*In Vitro*	—	Chen et al. (2017)

### Prostatic cancer

The incidence of prostate cancer ranks fourth among malignant tumors and second among male malignant tumors, second only to lung cancer (Cao et al., 2021; Zhu et al., 2021). The growth rate of prostate cancer ranks second among all tumors, and the mortality rate of male malignant tumors ranks the fifth (Lakkis and Osman, 2021; Hong et al., 2022). Conventional treatment of prostate cancer generally includes surgery, radiotherapy, chemotherapy and hormone therapy (Salaheldin et al., 2020; Wang et al., 2022b). However, despite the combination of successful active treatment and many traditional treatment, the annual mortality rate of prostate cancer has only slightly improved. The main reason is that radiotherapy and radical surgery will seriously reduce the quality of life of patients, while chemotherapy drugs and hormone therapy, due to lack of specificity, often produce many toxic and side effects when used in large doses, which seriously affect patients’ survival time (Liu et al., 2020b). Therefore, it is urgent to develop a targeted drug delivery system with specific recognition ability for prostate cancer, which can reduce the toxicity of drugs and enhance the therapeutic effect of drugs. This drug delivery system based on aptamer not only has specific targeting to tumor cells, but also has the advantages of controlled drug release, synergistic therapy and real-time monitoring of therapeutic effect (Wang et al., 2019), which gradually attracts people’s attention.

DOX is one of the most widely used anticancer drugs in tumor treatment. However, due to various side effects of these anticancer drugs, their clinical application are limited (Maurer et al., 2016). In order to reduce or even eliminate the toxic and side effects of DOX, scientists have made a series of efforts. Jing et al. designed a DOX delivery system mediated by double aptamer modified tumor targeting gene and recombinant adenovirus (A10–3.2 (DOX)/DUP-1-PEG-Ad5, ADDP-Ad5) (Jing et al., 2016). As polypeptide aptamer DUP-1 targeted prostate specific membrane antigen (PSMA) negative cells, while RNA aptamer A10–3.2 targeted PSMA positive prostate cancer cells, DOX delivery system can simultaneously targeted PSMA positive LNCaP and PSMA negative PC3 cells. *In vitro* experiment results showed that ADDP-Ad5 could effectively inhibit the activity of prostate cancer cells by releasing tumor suppressor genes phosphatase, tensin homologues and DOX, and obviously inhibited the growth of LNCaP and PC3 tumor transplanted tumors, it has no inhibitory effect on normal prostate cells and no obvious toxicity to mice. This experiment has been proved that ADDP-Ad5 provided an effective and powerful drug delivery platform for the effective treatment of cancer, and plays a guiding role in the development of tumor molecular targeted therapy. It has great potential in clinical application in the treatment of prostate cancer.

**FIGURE 7:**
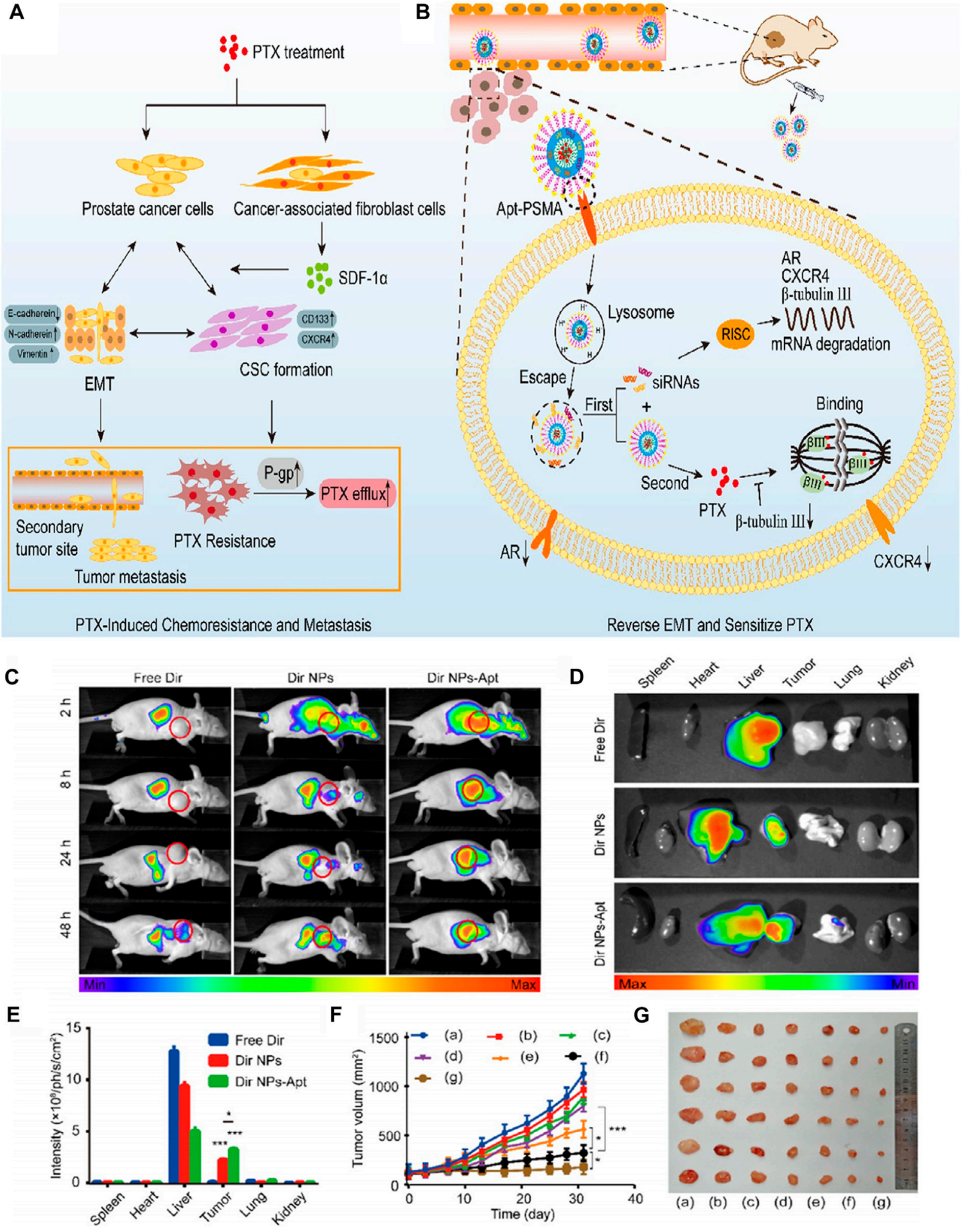
**(A)** PTX treatment is found to promote PTX resistance and metastasis *via* inducing EMT. **(B)** Reversing EMT by PTX/siRNAs NPs-Apt in PCa cells can sensitize PTX and inhibit metastasis. **(C)** Image of fluorescence distribution in nude mice bearing tumors after intravenous injection of free Dir, Dir NPs, or Dir NP-Apt. Fluorescence images **(D)** of nude mice and mean fluorescence intensity **(E)** of isolated tumor tissues and organs. **(F)** Tumor volume of different groups. **(G)** Image of tumor size. Reproduced with permission from Ref. (Guo et al., 2021), Copyright 2021, American Chemical Society.

Combination chemotherapy is an effective strategy for the treatment of prostate cancer. Lipidpolymer hybrid nanoparticles (LPN) are core-shell nanoparticles consisting of polymer core and lipid shell. As a drug delivery carrier, LPN provided significant advantages for the comprehensive therapeutics of prostate cancer. Chen et al. synthesized a multifunctional nanoplatform LPN (APT-CUR/CTX-LPNS) which was aptamer functionalized and can co-deliver curcumin (CUR) and azataxol (CTX) (Chen et al., 2020b). *In vitro* and *in vivo* experiments showed that APT-CUR/CTX-LPNs drug ratio of 2: 5 (CUR: CTX) showed good cell inhibition ability, high tumor aggregation rate and remarkable tumor inhibition effect. APT-CUR/CTX-LPN provided a good application prospect for dual administration of prostate cancer *in vivo*, showing the potential of synergistic combination therapy for prostate cancer. Paclitaxel (PTX) is a commonly used chemotherapy drug for the treatment of prostate cancer, but a large number of patients usually develop drug resistance after short-term therapeutics. Therefore, it is urgent to develop an effective treatment strategy to overcome PTX resistance and prostate cancer metastasis. To explored the combined therapy of PTX-resistant prostate cancer, Guo et al. established LNCaP/PTX cells resistant to PTX, and also synthesized PSMA aptamer functionalized shell core nanoparticles (PTX/siRNAs NPs-Apt). The shell was composed of calcium phosphate (CaP) adsorbing siRNAs and hydrophilic Apt-PEG2K, and the core is formed by coating PTX with hydrophobic phosphatidylethanolamine (DSPE) (Guo et al., 2021). After targeted delivery of PTX/siRNAs NPs-Apt to prostate cancer cells, siRNAs and PTX are released in turn, in which CaP can trigger lysosome escape, ensure that aggregated sirnas are efficiently released into cytoplasm, reverse epithelial mesenchymal transition (EMT), and sensitize PTX again, while PTX located in the core is then released, playing the killing role of chemotherapy to achieve the best synergistic effect (Figure 7). Experiments have been proved that PTX/siRNAs NPs-APT has specific tumor targeting and remarkable tumor inhibition effect in subcutaneous and *in situ* prostate cancer tumor models, and there is no obvious side effects *in vivo*. It is a promising nanodrug delivery system for the treatment of PTX-resistant prostate cancer.

## Conclusion

In the recent 20 years, aptamers have been widely used in biomedicine and materials science because of their unique recognition characteristics. AFNs have both aptamer targeting and high loading, biocompatibility and stability of nanomaterials, which have great potential application value in targeted therapy of tumors. Although some progress has been made in cell experiments and animal experiments, it should be pointed out that there are still great challenges in the application of AFNs in clinical diagnosis and treatment. In a word, we expect that future research on aptamers integrated nanomaterials will focus on the following aspects. First of all, it has good biocompatibility, high entrapment efficiency and responsiveness to intermediate release, and can be safely used in biological systems and *in vivo*. Secondly, a variety of aptamers or nanomaterials are integrated together to form a multifunctional nanosystems with complementary advantages, which can diagnose and treat various diseases at the same time. Finally, it can simultaneously load a variety of preparations, such as drugs, imaging agents, photosensitizers, fluorescent agents and gene drugs, and can monitor the therapeutic effect in real time at the same time of collaborative therapeutics, thus realizing the precise integration of diagnostics and therapeutics.
